# Implications of immune dysfunction on endometriosis associated infertility

**DOI:** 10.18632/oncotarget.12577

**Published:** 2016-10-11

**Authors:** Jessica E. Miller, Soo Hyun Ahn, Stephany P. Monsanto, Kasra Khalaj, Madhuri Koti, Chandrakant Tayade

**Affiliations:** ^1^ Department of Biomedical and Molecular Sciences, Queens University, Kingston, Ontario, Canada

**Keywords:** inflammation, cytokines, oxidative stress, infertility, endometriosis

## Abstract

Endometriosis is a complex, inflammatory disease that affects 6-10% of reproductive-aged women. Almost half of the women with endometriosis experience infertility. Despite the excessive prevalence, the pathogenesis of endometriosis and its associated infertility is unknown and a cure is not available. While many theories have been suggested to link endometriosis and infertility, a consensus among investigators has not emerged. In this extensive review of the literature as well as research from our laboratory, we provide potential insights into the role of immune dysfunction in endometriosis associated infertility. We discuss the implication of the peritoneal inflammatory microenvironment on various factors that contribute to infertility such as hormonal imbalance, oxidative stress and how these could further lead to poor oocyte, sperm and embryo quality, impaired receptivity of the endometrium and implantation failure.

## INTRODUCTION

Endometriosis is a chronic, inflammatory, estrogen-dependent disease that is characterized by the growth of endometrial tissue outside of the uterine cavity. Endometriosis affects 6-10% of reproductive-aged women. Despite its staggering economic impact ($1.8 Billion/year in Canada [1]; $22 Billion/year in the USA [2]), endometriosis remains misdiagnosed, misunderstood and ineffectively treated. The cause of this enigmatic disease is unknown; however, the theory of retrograde menstruation is widely accepted. This theory suggests that endometrial tissue, sloughed off during menstruation, is refluxed into the fallopian tubes and peritoneal cavity during menstrual uterine contractions. However, 76-90% of women have been shown to experience retrograde menstruation [3]. This has prompted researchers to question why only 6-10% of women develop endometriosis if so many women experience retrograde menstruation. To date, it has been suggested that the women who develop endometriosis have genetic, biochemical, or immunological dysfunction that prevents the removal of the tissue from the peritoneal cavity and rather facilitates tissue adhesion to peritoneal structures [4].

It has been well established that the immune system of women with endometriosis is dysfunctional. A multitude of immune cell types, including neutrophils, macrophages, dendritic cells, natural killer cells, T helper cells and B cells have been shown to be dysregulated in women with endometriosis [5–10]. Additionally, cytokines and chemokines involved in inflammation, angiogenesis and tissue growth are increased in the plasma and peritoneal fluid (PF) of women with endometriosis [11, 12]. This local and systemic inflammatory *milieu* is suspected to stimulate symptoms commonly presented including pain and infertility [13, 14]. It has been reported that 35-50% of endometriosis patients experience infertility and 25-50% of infertile women have endometriosis [15]. The monthly fecundity rate in healthy couples, which is a couple's probability of conceiving in one month, is 15-20% [16]. In contrast, women with endometriosis have a monthly fecundity rate of 2-10% [17]. This suggests that women with endometriosis have a significantly lower likelihood of conceiving each month. While the use of medical therapeutics including contraceptive steroids, progestins, aromatase inhibitors, agonists of gonadotropin-releasing hormone and non-steroidal anti-inflammatory agents are helpful to relieve pain, the use or ceasing use of these agents rarely improves fertility [18–20]. Additionally, the side effects of long term use can be detrimental; for example, the use of oral contraceptives has been show to affect endometrial thickness and growth [21]. Due to improved fecundity observed during randomized control trial studies [22, 23], surgical removal of an endometriotic lesion and assisted reproductive technologies are used to treat endometriosis associated infertility. However, why surgical excision of endometriotic lesions improves fertility is unknown. It has been suggested that surgical removal of endometriotic lesions decreases peritoneal inflammation and results in improved fertility. Previous reports from our group have demonstrated that plasma and PF concentrations of inflammatory cytokines such as GM-CSF, IL-2, IL-8, IL-10 and IL-17 significantly decreased following lesion excision, suggesting inflammation is prompted by the lesion [24, 25]. These findings indicate the probable impact of endometriosis associated inflammation towards infertility.

Not only is the pathogenesis of endometriosis complex and unknown, the pathogenesis of infertility associated with endometriosis remains elusive. Furthermore, why some women with endometriosis display infertility and others do not is unknown. Infertility is not uniformly associated with disease state or size of the lesion, which prompts researchers to seek other conclusions to explain how infertility manifests in these patients. Immune system dysfunction is speculated to play a large role in endometriosis associated infertility; however, its precise role is rarely discussed in depth. In this review, we compile and analyze the current literature, including our own work, surrounding the role of the inflammation and immune system dysfunction in women with endometriosis associated infertility.

## INFLAMMATION, IMMUNE DYSFUNCTION AND INFERTILITY

The pathogenesis of infertility associated with endometriosis is complicated by the involvement of biochemical, endocrine, immune and genetic factors. Whether immune dysfunction initiates the pathogenesis of endometriosis or is a product of the disease has not been identified. After decades of research, there appears to be a consensus that the immune system of women with endometriosis and women with endometriosis associated infertility is different from healthy, fertile controls. Both cellular and secreted immune mediators are aberrantly expressed in the PF and plasma of endometriosis patients. Pro-inflammatory cytokines such as TNF-α, IL-1 β, IL-6, IL-8, IL-10, IL-17, IL-33, IP-10, MCP-1 MIF and RANTES are aberrantly expressed in the PF of women with endometriosis [25–32]. Additionally, women with endometriosis associated infertility have an altered intraperitoneal immune cell status compared to women with unexplained infertility [33]. However, most of these published studies are observational and do not provide mechanistic pathways.

In the pursuit of understanding infertility in endometriosis, molecular profiling has been used to identify differential gene expression in infertile, endometriotic patients. Using Nanostring immune transcriptomic profiling of 539 immune-inflammation related genes, our group found 91 genes that were aberrantly expressed in the eutopic endometrium of infertile, endometriosis patients. The differentially expressed genes were predominantly involved in cellular adhesion, cytokine-cytokine interaction, apoptosis and decidualization [34]. Specifically, pro-inflammatory cytokines, chemokines and receptors including *CXCL1, CX3CL1, CXCL9, CXCL10, IL-32, CXCR2, IL-7R* and adhesion molecules including *ICAM3* and *SELL* had a higher expression in the eutopic endometrium of infertile, endometriosis patients compared to fertile controls [34]. Furthermore, a decreased expression of *NOTCH1* and *NOTCH2* was observed, which aligns with previous studies associating disturbed NOTCH pathway signaling with impaired decidualization in the endometrium of women with endometriosis [34, 35]. In another study, a number of proteins important for inflammation and oxidative stress, including sphingosine kinase A, Hypoxia inducible factors, heat shock cognate proteins and superoxide dismutase, were found to be dysregulated in the eutopic endometrium from infertile women with ovarian, stage IV endometriosis [36]. The evidence shown here indicates inherent differences in the eutopic endometrium from infertile women with endometriosis. It is plausible that chronic, peritoneal inflammation is involved in shaping the transcriptome of eutopic endometrium in patients.

Cellular components of the immune system are dysregulated in endometriosis patients and specifically infertile endometriosis patients. Uterine natural killer (uNK) cells are a subset of NK cells residing in the uterus and play important roles in pregnancy and the development of the placenta [37]. Uterine NK cells have been linked to spiral artery remodeling and produce pro-inflammatory cytokines such as GM-CSF, CSF-1, TNF-α, IFN-γ, TGF-β, LIF and IL-2 and these cytokines have been associated with invasion of trophoblasts into the uterine wall [38, 39]. Aberrant numbers of uNK cells are linked to pregnancy pathologies including pre-eclampsia and fetal growth restriction [40]. Increased infiltration of CD16+ cytotoxic uNK cells, are found in the eutopic endometrium of women with endometriosis associated infertility [41]. Additionally, healthy, fertile controls have more mature uNK cells; however, immature uNK cell populations exist in infertile women with endometriosis [42]. Further studies are required to understand exactly why uNK cells are not maturing in infertile endometriosis patients and how this affects fertility as a whole.

T regulatory (Treg) cells are altered in endometriosis patients and have been suggested to play a role in the pathogenesis of endometriosis and its associated infertility [43]. Lower numbers of Treg cells have been detected in the eutopic endometrium of a non-human primate endometriosis model [44]. Interestingly, in humans, the expression of the transcription factor forkhead P3 (Foxp3), a distinctive surface marker for Treg cells, is upregulated in the endometrium of women with endometriosis [45]. Additionally, higher expression of FoxP3 mRNA has been detected in the endometrium of infertile, advanced stage endometriosis women; that said, the Foxp3 protein was not significantly higher in these patients compared to fertile controls [46]. Furthermore, TGF-β has been shown to promote differentiation and induction of Treg cells [47]. TGF-β has been shown to be upregulated in the PF of women with endometriosis [48], which indicates that aberrant expression of Treg cells could be due to aberrant concentrations of TGF-β in the PF of these women. In contrast, women with unexplained infertility have shown reduced expression of Foxp 3 is in the endometrium of compared to healthy fertile controls [49]. While the precise mechanism linking Treg cells and infertility requires further investigation, previous reports studying the pre-implantation period in murine pregnancy indicate that Treg cells play a critical role in implantation [50]. The presented evidence suggests that dysregulation of Treg cells may contribute to implantation failure observed in endometriosis patients.

There are other important immune cell types including macrophages and dendritic cells associated with inflammation in endometriosis. While it is likely that these cell types are involved in the endometriosis associated infertility, studies directly associating them with infertility in patients are scarce within the literature. Nevertheless, more studies are required to delineate coordinated interactions between various immune cells in the promotion and/or resolution of inflammatory cascade and its impact on infertility in endometriosis patients.

## IMPLICATION OF IMMUNE DYSFUNCTION ON THE ENDOCRINE/IMMUNE PATHWAY

A specific and delicate balance between inflammatory factors and sex-hormones are required for the initiation and propagation of normal female reproductive events including folliculogenesis, ovulation, menstruation, embryo implantation and pregnancy. Disruptions in the endocrine/immune pathway, such as inflammation from endometriosis, create deleterious effects on reproductive function.

It is now well established that the growth of endometriotic lesions is dependent on estrogen. It has been suggested that estrogens are delivered to the ectopic lesions in an endocrine fashion; however, new evidence suggests that endometriotic lesions produce estrogen themselves [51]. This feed forward loop creates myriad of cell signaling cascades in the peritoneal microenvironment. Aromatase P450 (aromP450) is important for the production of estrogen as this enzyme catalyzes the reaction to produce estrone from androsteneione and 17B-hydroxysteroid dehydrogenase type 1 which is catalyzed into the biologically active form of estrogen, estradiol (E2) [14]. Aromatase P450 is significantly upregulated in the eutopic endometrium of women with endometriosis compared to healthy controls and aberrantly high expression is consistent regardless of disease state [52]. This suggests that in endometriosis patients, both the eutopic and ectopic endometrial tissue are involved in excess production of estrogen further. Additionally, in the presence of growth factors and pro-inflammatory cytokines including IL-1 β, TNF-α, IFN-γ and IL-17, cyclooxygenase-2 (COX-2) is induced and COX-2 catalyzes the synthesis of prostaglandin E_2_ (PGE_2_). Production of prostaglandins and cytokines have been suggested to facilitate infertility in women with endometriosis [19]. Increased expression of COX-2 mRNA is observed in the ectopic and eutopic endometrium of women with endometriosis compared to healthy controls [52]. E2 has been shown to stimulate COX-2 expression, which suggests that a positive feedback loop exists [53]. Together, aberrant expressions of COX-2 and aromP450 produce a local, continuous stream of E2 and PGE_2_ in endometriosis patients [52, 54] creating a state of estrogen dominance [14]. Estrogen, in particular, plays a critical role in female reproductive events including oocyte maturation, fertilization, ovulation and implantation. Estrogen initiates proliferation and differentiation of granulosa cells and facilitates the actions of luteinizing hormone and follicle stimulating hormone, which are important for folliculogenesis and ovulation [55]. Estrogen also stimulates growth of the uterine lining, which is important for uterine receptivity and implantation of a fertilized embryo at specific time periods. As such, continual high expression of estrogen likely interferes with the transition from proliferative to secretory phase and other important reproductive events. A state of estrogen dominance is also detrimental as estrogen has been shown to be an inhibitor of αvβ3 integrin, a critical marker for endometrial receptivity, in the uterine lining [56]. When analyzing fertility, the receptivity of the endometrium is a critical factor for successful attachment, implantation and pregnancy [57] and therefore, inhibition of integrins and adhesion molecules that facilitate receptivity likely disturbs fertility.

In addition to aberrant production of sex-hormones, the aberrant expression and signalling of the progesterone receptor (PR) has been demonstrated in women with endometriosis [14]. A high expression of the decoy receptor, progesterone A receptors (PRA), relative to progesterone B receptors (PRB) has been found in the eutopic endometrium of women with endometriosis [58]. Reduced expression of PRB prevents appropriate progesterone signalling and this progesterone resistance has been categorized as a “hallmark for implantation failure” since progesterone facilitates decidualization [14]. Emerging evidence suggests that expression of both estrogen receptors (ER) and PR are altered by inflammation. Specifically, aberrant expression of ER and PR has been associated with an overexpression of IL-1, IL-6 and TNF-α [59]. Grandi et al demonstrated that TNF-α and IL-1β (upregulated in the PF of women with endometriosis) cause a decrease in the expression of PRA and PRB mRNA in endometrial stromal cells isolated from women with endometriosis [60]. Additionally, Heublein et al demonstrated that TNF-α down regulates the expression of G-protein coupled estrogen receptor (GPER) in endometrial stromal cells isolated from women with endometriosis and the presence of GPER has been suggested to act as a selector that is important for folliculogenesis and follicle maturation [61]. The presented evidence demonstrates not only that aberrant expression of PR and ER are correlated with an increase in inflammatory mediators but that inflammation can directly alter the expression of both PR and GPER.

While treating endometriosis patients using hormonal intervention was previously emphasized, the regulation of inflammation and hormones appear to be interconnected and complex. Therefore, a comprehensive understanding of immune dysfunction in endometriosis patients is of the utmost importance to treat endometriosis and endometriosis associated infertility.

## IMPLICATION OF IMMUNE DYSFUNCTION ON OOCYTE QUALITY, SPERM MOTILITY AND EMBRYOTOXICITY

Retrospective analyse of *in vitro* fertilization (IVF) and oocyte donation programs consistently find that women with endometriosis have significantly reduced pregnancy rates per cycle and per transfer as well as reduced implantation rates [62–64]. They also find that healthy ovum donation to endometriosis patients produces the same rate of implantation and pregnancy compared to controls [62–64]. Additionally, retrospective and prospective clinical trials using IVF have shown decreased oocyte and embryo quality and low ovarian reserves in women with endometriosis compared to controls [65, 66]. Collectively, human studies indicate poor oocyte and embryo quality and lower pregnancy rates in women with endometriosis.

Because the female reproductive organs, including the uterus, ovaries and fallopian tubes, are bathed in PF, pro-inflammatory cytokines and chemokines interact with the oocyte and embryo, which can impact growth and can inflict damage to the oocyte and embryo. A damaging inflammatory *milieu* in endometriosis patients offers a plausible theory to explain why the quality of oocytes and embryos is lower. Intra-follicular levels of IL-8, IL-12 and adrenomedullin are elevated in women with endometriosis undergoing IVF and are indicators of impaired embryo and oocyte quality [67]. In retrospective IVF studies, poor oocyte quality was observed and measured by diminished blastomere cleavage rates, increased numbers of arrested embryos and impaired cytosolic events [68–71]. Sperm, travelling through the uterus and fallopian tubes, also interact with inflammatory cytokines in the PF and similarly encounter damage. Inflammatory cytokines including TNF- α and oxidative stress has been shown to directly hinder sperm motility [72]. Similarly, murine embryos incubated in the PF from women with endometriosis have shown diminished growth rates of embryos, increased rates of apoptosis, DNA fragmentation and increased number of embryos arrested in development [73–76]. Dexamethasone reduced the observed embryotoxic effect of the PF from women with endometriosis associated infertility [77]. Dexamethasone is a glucocorticoid that has been shown to reduce the expression of prostaglandins and other inflammatory mediators dysregulated in endometriosis [78]. Additionally, inhibiting TNF-α reduces embryotoxic effect on mouse embryos incubated with PF from infertile women with endometriosis [79]. Collectively, these studies link inflammation in the PF, specifically TNF-α, with embryo toxicity. Studying the toxicity of PF from women with endometriosis is limited by ethical constraints as interfering with human embryos violates moral and ethical considerations. However, this murine model provides a convincing argument to suggest the PF from women with endometriosis produces a damaging effect on the embryo.

## IMPLICATION OF IMMUNE DYSFUNCTION ON OXIDATIVE STRESS

An imbalance between oxidants and antioxidants creates oxidative stress [80]. This ratio can be altered by an increase in the expression of oxidants such as reactive oxygen species (ROS) and reactive nitrogen species or by a decrease in the expression of antioxidants such as superoxide dismutase, glutathione or vitamin E [81]. Many inflammatory diseases have been correlated with high oxidative stress including rheumatoid arthritis, cardiovascular disease, fibrosis and pre-eclampsia [82–85]. An increase in pro-inflammatory cytokines generates ROS and induces apoptosis or necrosis by activating transcription factors such as AP-1, p53 and NF-κB [81]. In addition, estrogen and metabolites of estrogen have been shown to generate ROS, which have been shown to contribute to breast cancer [86, 87].

Higher levels of oxidative stress markers have been observed in in the PF of women with endometriosis specifically, high concentrations of malondialdehdye (MDA) and oxidized low density lipoprotein [88]. Elevated concentrations of a lipid peroxidation metabolite, 8-iso-prostaglandin F2-alpha, has also been detected in the PF and urine of women with endometriosis and has been speculated to play a role in infertility associated with endometriosis [88]. In another study, nine infertile women with endometriosis were shown to have an increased concentration and increased activity of nitric oxide synthase (NOS), an enzyme found in peritoneal macrophages, compared to fertile controls [89]. Other investigators have failed to find increased oxidative stress markers in the PF of women with endometriosis [90]. Why we observe such differences is unknown. Similarly, high concentrations of oxidative stress molecules and low levels of antioxidative molecules have been found in the follicular fluid from women with endometriosis. Singh et al detected higher levels of ROS, NOS and MDA in the follicular fluid of women with endometriosis and another study detected an increased concentration of 8-hydroxy-2’-deoxyguanosine, a metabolite of DNA oxidative damage, in the follicular fluid of specifically infertile women with endometriosis [91, 92]. In addition, the concentrations of critical antioxidants are aberrantly expressed in endometriosis patients. Levels of glutathione are diminished in the follicular fluid of women with endometriosis [93] and low levels of other antioxidants including vitamin A, C and E were found to be significantly reduced in the intra-follicular fluid of women with endometriosis associated infertility compared to women with tubal infertility [92]. As expected, higher levels of glutathione have been correlated with higher quality embryos [93]. Correspondingly, high concentrations of oxidative stress metabolites and lower concentrations of antioxidant molecules are associated with less successful IVF treatments [92].

Oxidative stress offers a plausible mechanism to link inflammation and infertility as IL-1β and TNF-α are able to activate apoptotic mechanisms [81] and oocytes from women with endometriosis have exhibited increased rates of apoptosis in the cumulus cells [94]. Ovarian cell apoptosis is used as an indicator for low oocyte quality [95] and therefore suggests that immune induced oxidative stress may be directly deteriorating oocyte quality.

## THERAPEUTIC INTERVENTION: TARGETING INFLAMMATION AND IMMUNE DYSFUNCTION

After twenty years of research, unfortunately, no new treatments have come to the market to relieve the symptoms of endometriosis. While inhibiting inflammatory cytokines, specifically TNF-α had shown promise, anti-TNF-α treatment did not improve fertility in a non-human primate model and a clinical trial in human patients inhibiting TNF-α did not provide pelvic pain relief compared to a placebo [96, 97]. More recently, inhibition of the mitogen activated protein kinase (MAPK) cascade has emerged as a potential therapeutic for endometriosis, as it is activated by pro-inflammatory cytokines and oxidative stress and causes recruitment of immune cells to amplify the inflammation [54]. Sorafenib and Vemurafenib are different inhibitors of the MAPK cascade and have shown therapeutic promise both *in vitro* and in a mouse model by targeting endometriosis related inflammation [98, 99]. Human clinical trials of Sorafenib have been conducted and are underway for treatment of cancers including hepatocellular carcinoma, bladder cancer, acute myeloid leukemia (www.clinicaltrials.gov). Vermurefenib was used in human clinical trials for the treatment of advanced thyroid cancer and melanoma (www.clinicaltrials.gov). However, to our knowledge, human clinical trials using Sorafenib or Vemurafenib for the treatment of endometriosis have not been attempted. Many other inhibitors exist for a variety of targets in the MAPK cascade with the intention to reduce inflammation, proliferation and angiogenesis through cytokines and chemokines; however, these seem, to our knowledge, to be in the early stages of research and development [54]. Overall, the upstream regulatory factors of inflammatory cascades are emerging as the next therapeutic targets for endometriosis and endometriosis associated infertility.

## CONCLUSIONS AND FUTURE DIRECTIONS

**Figure 1:**
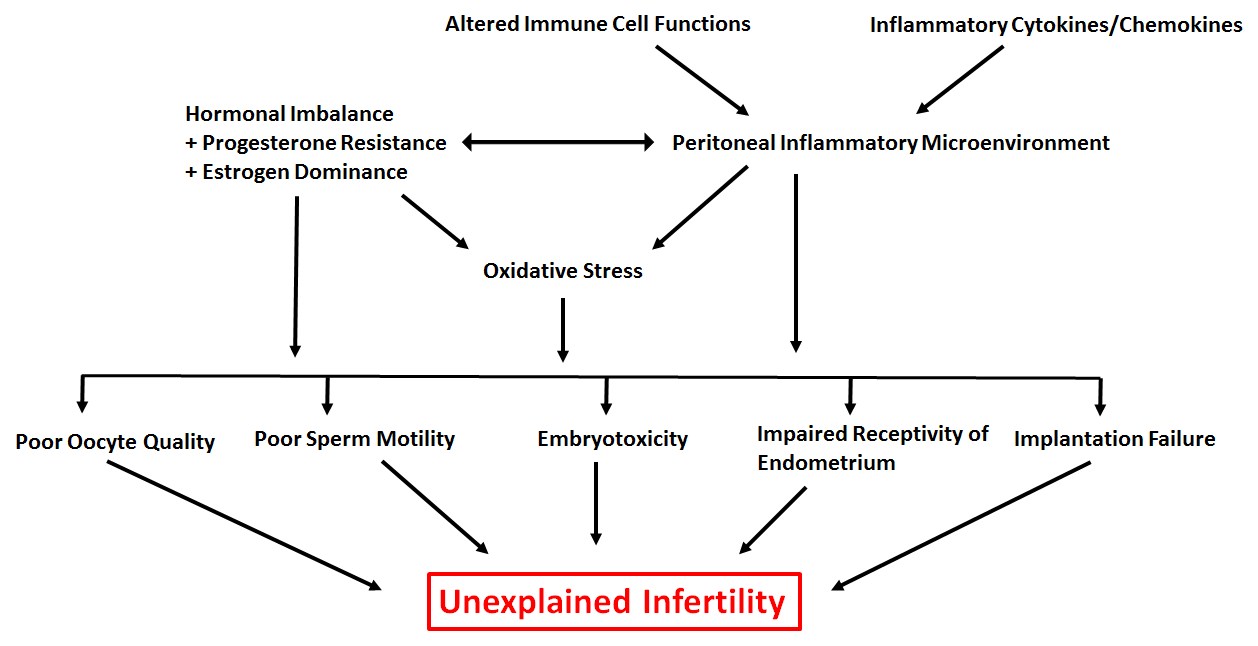
Overview of the underlying contributors to unexplained infertility associated with endometriosis Hierarchical arrows indicate a potential link between various factors contributing to unexplained infertility associated with endometriosis.

Here we provided evidence from the literature and from our own work to understand how the immune system is potentially dysfunctional in endometriosis patients with infertility issues. We also provided insights into the implications of this dysfunction (summarized in Figure 1). There is substantial evidence to suggest that aberrant immune mechanisms in endometriosis are associated with a number of factors that have the ability to deteriorate fecundity including folliculogenesis, oocyte and embryo quality and eutopic receptivity/implantation failure. However, it remains unclear how immune dysregulation is contributing towards pathogenesis of this enigmatic disease. At this time, it is also unclear if a disruption of one, some or all factors lead to infertility. As stated earlier, there has been limited progress in terms of new treatment strategies to manage endometriosis related infertility. Further understanding of the mechanisms and complex interplay between immune-endocrine axis may help explain why we observe heterogeneity of the symptoms in patients and may stimulate the process of developing a more comprehensive and accurate classification system.

Previous therapies for endometriosis have targeted downstream, end products such as levels of estrogen, TNF-^α^ and COX-2. Unfortunately, these therapeutics fail to uniformly reduce symptoms, specifically infertility, and fail to cure the disease. Targeting upstream, regulatory mediators seems rational for future action. Many studies correlate endometriosis and its associated infertility with an up or down regulation of immune products; however, few analytical and explanatory studies exist with the objective to understand why we observe immune dysfunction. Are the increased numbers of infiltrating cell types (if any) in the peritoneal cavity the problem or their functional incompetence for example activation status or cytotoxicity causing dysfunction? Additionally, the complex connection between inflammation and the hormonal imbalance observed is poorly understood. Investigators studying endometriosis seem to have exhausted intuitive explanations to understand the pathogenesis of endometriosis and its associated infertility. Therefore, there is a pressing need at this time in endometriosis research, similar to other chronic inflammatory conditions including cancer, to shift the focus of investigation to understand the complex immunological pathways leading to the disease in order to treat and cure endometriosis along with its infertility.
